# The Role of Adria Plate Lithospheric Structures on the Recent Dynamics of the Central Mediterranean Region

**DOI:** 10.1029/2021JB022377

**Published:** 2021-10-08

**Authors:** Rosalia Lo Bue, Manuele Faccenda, Jianfeng Yang

**Affiliations:** ^1^ Dipartimento di Geoscienze Università di Padova Padova Italy

**Keywords:** Central Mediterranean, Adria plate, seismic anisotropy, shear wave splitting, subduction zone processes

## Abstract

The Tertiary tectonic evolution of the Central Mediterranean has been relatively well constrained by abundant geological data. Yet, several uncertainties persist about the mechanisms that led to the present‐day surface morphology and deep slab geometry. Here, we combine geodynamic and seismological numerical modeling techniques to reproduce the recent large‐scale evolution of the Central Mediterranean and the associated strain‐induced upper mantle fabrics and seismic anisotropy. 3D thermo‐mechanical subduction models were designed and calibrated according to paleogeographic‐tectonic reconstructions and seismological observations available in the literature. It is found that, although the opening of back‐arc extensional basins in response to the retreat of the Ionian slab is a common feature in all models, structural heterogeneities within the Adria plate and/or the geometry of its Tyrrhenian passive margin profoundly impact on the segmentation of the subducting slab and the amount of Ionian trench retreat. More, in general, this study highlights the importance of coupling geodynamic and seismological modeling to better constrain the tectonic evolution of complex convergent margins such as the Central Mediterranean.

## Key Points

- Geodynamic and seismological modeling methods are combined to reproduce the recent evolution of the Central Mediterranean
- The influence on trench shape and slab tears of structural heterogeneities within the Adria plate is addressed
- Geodynamic models are validated against seismic anisotropy observations

## 1 Introduction

In the geodynamic context of the slow convergence between the African and Eurasian plates, the Central Mediterranean region has been involved in a complex subduction process that over the last 30 Myr was characterized by the rapid retreat of the Ionian trench, the opening of back‐arc extensional basins (i.e., Liguro‐Provençal, Algerian, and Tyrrhenian basins) and episodes of slab lateral tearing, segmentation, and break‐off (Carminati et al., 2012; Faccenna et al., 2004, 2007, 2014; Jolivet et al., 2006, 2009; Mauffret et al., 2004; Rosenbaum et al., 2002b; van Hinsbergen et al., 2014, 2020). Although the shallow tectonic evolution has been relatively well constrained by a wealth of geological data, several uncertainties persist about the recent mantle dynamics of this region and the interaction between surface tectono‐magmatic processes and deep mantle convection patterns. Analog and/or numerical modeling of 3D mantle flow evolution specifically designed for the Mediterranean could provide important clarifications about these processes. However, given the high complexity of the Mediterranean tectonic setting, few research groups have attempted to reproduce its evolution.

On the one hand, analog models of a single subducting and rolling back slab have been able to address and reproduce some of the key aspects of the Central Mediterranean history such as (a) the initiation of the subduction process, (b) the subduction dynamics and their relationships with plate kinematics, (c) the episodicity of back‐arc extension, and finally (d) the role played by slab‐induced mantle circulation (Funiciello & Faccenna, 2010; Guillaume et al., 2010, 2013). On the other hand, 3D numerical models have highlighted the importance of considering appropriate plate geometries and lateral buoyancy variations to reproduce the recent dynamics of the Central‐Western Mediterranean and Alpine regions (Kaus et al., 2020; Lo Bue & Faccenda, 2019; Magni et al., 2014).

Among the aspects of the Central Mediterranean geodynamic history that have not yet been fully clarified, there are (a) the development and evolution of the episodes of slab tearing and segmentation along northern Africa and the western Adria plate margins, and (b) the relations between mantle dynamics and plate surface and deep kinematics. The latter can be estimated by measuring seismic anisotropy generated by strain‐induced lattice/crystal preferred orientation (LPO/CPO) of intrinsically anisotropic minerals, which is thought to be the primary source of seismic anisotropy (Park & Levin, 2002). As there is a direct link between upper mantle deformation and macroscopic seismic anisotropy, a quantitative analysis of the seismic anisotropy may help to reconstruct the recent upper mantle flow patterns.

Laboratory experiments indicate that the strain‐induced LPO patterns vary as a function of the deformation history, temperature, deviatoric stress, and water content conditions (Karato et al., 2008). It follows that the extrapolation of the mantle flow from seismic anisotropy is neither simple nor always warranted, especially at subduction zones where complex and non‐steady‐state 3D flow patterns may establish. A promising approach that helps to reduce the number of plausible models that can explain a given anisotropy data set is to compare seismic measurements with predictions of numerical and experimental flow models (Long et al., 2007). Traditional numerical modeling studies of mantle fabrics and the associated anisotropy assume time‐independent, steady‐state flow dynamics (Becker, 2006). This method is effective in global intra‐oceanic contexts (e.g., Pacific and Atlantic oceans) where plate motions and shallow mantle flow remained stable over the past 43 Ma (Faccenna et al., 2013). Recently, Faccenda and Capitanio (2012, 2013) have extended this methodology to account for the non‐steady‐state evolution typical of many subduction zones, yielding mantle fabrics that are physically consistent with the deformation history. Hu et al. (2017), Zhou et al. (2018), and Confal et al. (2018) applied a similar modeling approach to real tectonic settings, such as the South America, the North America, and the eastern Mediterranean subduction systems, respectively.

In this study, we first briefly review the Central Mediterranean tectonic evolution and the seismological data available for the region. Subsequently, we show the results of applying the modeling methodology of Faccenda and Capitanio (2012, 2013) to the Central Mediterranean convergent margin. The modeling results are then evaluated and discussed by comparing seismological synthetics (SKS splitting, Rayleigh and P‐wave anisotropy, and isotropic anomalies) and the predicted tectonic evolution with geophysical and geological observations. Throughout the study, we use “Ma” to indicate geological time before present, and “Myr” when referring to the elapsed time after the start of the model.

### 1.1 Tectonic Evolution of the Region

The tectonic evolution of the entire Mediterranean region in the Mid‐Late Cenozoic is connected with the relative motion of three main plates (Africa, Adria, and Europe) and in its central portion is dominated by the collision between the Adria and Eurasian plates along the Alps. Two oceanic trenches flanked the Alpine collision: the Liguro‐Provençal/Tyrrhenian trench on the western side (object of this study) and the Hellenic trench on the eastern side. The presence of an unknown number of smaller continental blocks and oceanic basins in the Tethyan realm produced intermittent phases of subduction and collision that led to the currently observed complex geological setting (Carminati et al., 2012; Faccenna et al., 2014).

**Figure 1:**
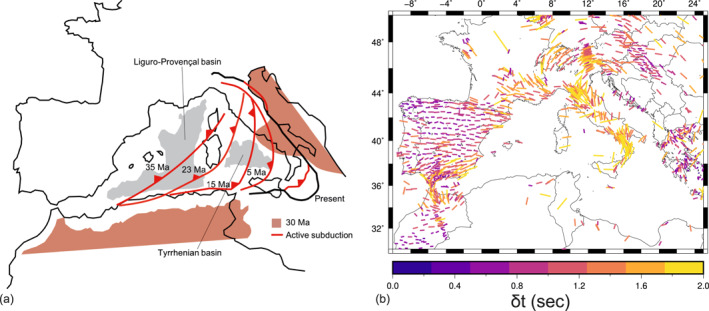
(a) Evolution of the Central Mediterranean region in the last 30 Myr (adapted from Lucente & Speranza, 2001, Lucente et al., 2006, Faccenna et al., 2014, and van Hinsbergen et al., 2014). Red lines indicate active subduction at different age stages, while the black thick line is the present‐day frontal thrust. The brown region illustrates the geometry of the Africa and Adria continental plates, excluding the thin margins, at 30 Ma (adapted from Lucente & Speranza, 2001, Lucente et al., 2006, and van Hinsbergen et al., 2014). (b) SKS‐splitting data in the Central‐Western Mediterranean (Becker et al., 2012) and references therein updated as of December 6, 2020 (http://www-udc.ig.utexas.edu/external/becker/sksdata). The bars show the orientation of the SKS fast component and their length and color‐coding is proportional to the delay time.

Before ∼30–35 Ma, the underthrusting of the European continental lithosphere below Apulia‐Adria induced the formation of the Alps. After ∼30–35 Ma, when the subduction rate of the Ionian ocean overcame the Africa‐Eurasia convergence rate, the tectonic regime along the oceanic trenches switched from compressional to extensional. The evolution of this later tectonic stage (Figure 1a) can be summarized in several episodes of slab roll‐back and opening of back‐arc basins (Carminati et al., 2012; Dewey et al., 1989; Gueguen et al., 1998; Faccenna et al., 2004, 2007, 2014; Jolivet et al., 2009; Malinverno & Ryan, 1986; Rosenbaum et al., 2002a; Wortel & Spakman, 2000).

Several widely accepted tectonic reconstructions indicate that at ∼35–30 Ma an incipient Ionian slab was already present in the upper mantle below the Corsica‐Sardinia continental block, which may have triggered the subsequent “spontaneous” evolution of the subduction system (Faccenna et al., 2001, 2014). In the first stage of back‐arc extension, from ∼32–30 Ma to ∼16–15 Ma, Corsica and Sardinia were separated from the European mainland by a ∼40° clockwise rotation, leading to the opening of the Liguro‐Provençal basin (Faccenna et al., 1997, 2014; Gattacceca & Speranza, 2002; Rollet et al., 2002; Rosenbaum et al., 2002b). The collision between the Corsica‐Sardinia microplate with the western margin of Adria caused the formation of the Apennines (Patacca et al., 1990).

The second major stage of trench retreat, from ∼15 Ma to the present‐day, led to the opening of the Tyrrhenian Sea basin. The first period of extension was characterized by continental lithosphere thinning in the northern Tyrrhenian Sea, and by trench retreat in the western part of the southern Tyrrhenian Sea. In the latest Messinian (6–5 Ma), the ultrafast roll‐back rate of the narrow Ionian slab toward the southeast induced the opening of the southern Tyrrhenian Sea basin and the formation of new oceanic lithosphere. This happened in two distinct episodes with the formation of the Vavilov Basin (4–3 Ma) and, further to the east, of the Marsili Basin (2–1 Ma) surrounded by the currently active Aeolian island arc (Faccenna et al., 2007).

### 1.2 Isotropic Seismic Tomographies

Since the late 1990s, seismic tomographic methods have revealed several positive velocities anomalies interpreted as subducted lithospheric portions lying below the Alboran, Kabylides, and Calabria‐Apennine areas (Bezada et al., 2013; Gutscher et al., 2002; Piromallo & Morelli, 2003; Spakman, 1991; Spakman et al., 1993; Spakman & Wortel, 2004; Van der Meer et al., 2018; Wortel & Spakman, 2000; Wortel et al., 2009).

A dominant feature in P‐wave tomographic models is a broad high velocity anomaly associated with the North Apenninic‐Calabrian slab (Amato et al., 1993; Lucente et al., 1999; Piromallo & Morelli, 2003; Selvaggi & Chiarabba, 1995; Spakman et al., 1993; Spakman & Wortel, 2004; Van der Meer et al., 2018).

To the south, the narrow Calabria slab appears to broaden at the base of the upper mantle and to stagnate in the mantle transition zone of the Central Mediterranean (Neri et al., 2009; Spakman & Wortel, 2004). A similar result was obtained by Giacomuzzi et al. (2012) using a tomographic inversion based on analysis of relative residuals of S‐wave arrivals from teleseismic events, and by Scarfì et al. (2018), who detected a narrowing of the subduction system caused by horizontal tearing affecting both the sides of the slab. Neri et al. (2012) analyzed different geophysical data sets (gravity anomalies, seismic tomographic structure, and seismicity within the crust and uppermost mantle) and proposed that the slab is still continuous beneath the central part of the Calabrian arc, whereas detachment has already occurred beneath the arc edges (beneath northern Calabria and northeastern Sicily). Presti et al. (2019) suggested that clear necking of the foundering slab has already occurred (at a depth of 150 km), slab detachment has not happened yet but appears to be geologically close, implying the imminent end of the subduction process in the Central Mediterranean.

Further north along the Apenninic chain, several seismic tomography studies associated high‐velocity anomalies to the subducted Ionian ocean and western margin of the Adria plate (Benoit et al., 2011; Giacomuzzi et al., 2012; Kästle et al., 2018; Koulakov et al., 2009; Lucente et al., 1999; Piromallo & Morelli, 2003; Rosenbaum et al., 2008; Spakman & Wortel, 2004; Van der Meer et al., 2018; Zhao et al., 2016). The same studies indicate that the high‐velocity anomalies found below the Calabrian arc and the Northern Apennines appear to be laterally continuous below 250 km depth. At shallower depths, instead, a slow velocity anomaly is found below central Italy, which is associated with the presence of a slab window.

The vertical continuity and lateral extent of the slab beneath the northern Apennines are debated. Based on early tomographic models showing no apparent connection between the Adriatic plate and the subducted lithosphere, Spakman (1990) and Spakman et al. (1993) interpreted the northern Apennines deep anomaly as a detached slab under the entire Apennines resulting from the lateral migration of a slab tear from north to south. Conversely, the subsequent regional mantle models of Piromallo and Morelli (1997, 2003) exhibit local evidence for a continuous slab beneath Tuscany, at the same location where Spakman et al. (1993) found a small slab gap only about 50 km wide. Other teleseismic tomography studies of the Italian peninsula do not reveal any significant gap in the subducted lithosphere below the northern Apennines, and suggest the presence of a slab window below the central‐southern Apennines and in between the two high‐velocity bodies in the northern Apennines and Calabrian regions (Amato et al., 1993; Lucente et al., 1999). Following these results, Lucente and Speranza (2001), proposed an evolution of the Apennines arc‐trench system strongly influenced by the irregular shape of the Adria continental margin colliding with the trench. According to this study, the cessation of subduction and the detachment of the slab beneath central Italy was possibly caused by the presence of a thick continental lithosphere promontory of the Adria plate. However, more recent studies suggest that the northern Apennines slab reaches a depth of only ∼150–300 km (Giacomuzzi et al., 2012; Spakman & Wortel, 2004).

El‐Sharkawy et al. (2020) used Rayleigh wave tomography to image the entire Mediterranean upper mantle down to about 300 km. Although Rayleigh wave tomographies are characterized by a low lateral resolution and are affected by vertical smearing (strictly speaking, also teleseismic body wave tomographies of the upper mantle bear the same problem), they provide a useful source of information about the structure of shallower mantle layers that supplement those derived from body wave models. The El‐Sharkawy et al. (2020)'s model highlights that the Calabrian slab is vertically continuous in the area beneath Calabria and easternmost Sicily but detached beneath the southern Apennines and the northern Sicily. In particular, a low‐velocity anomaly extending from 70 km down to about 250 km depth is detected beneath the central Apennines, which supports the presence of the Central Apennines Slab Gap. Below 250 km depth, a high‐velocity anomaly is detected below the central Apennines, which can be related to the top edge of the north‐westward dipping Calabrian Slab. Further north, the high‐velocity anomaly appears to extend down to only 250 km depth beneath the Northern Apennines.

### 1.3 Seismic Anisotropy

Seismic anisotropy is widespread in the Mediterranean and it shows a complex pattern that likely has some relations with the recent (20–30 Ma) tectonic evolution of this region (Buontempo et al., 2008; Lucente et al., 2006; Schmid et al., 2004). Measurements of shear wave splitting of core‐refracted (mainly SKS and SKKS, herein referred for simplicity to just SKS) waves in the Central Mediterranean is characterized by average delay times of around 1–2 s, suggesting the presence of well‐developed upper mantle fabrics and strong seismic anisotropy. The SKS fast azimuths have a preferential trend of NNW‐SSE (i.e., trench parallel) along the Apenninic belt, which is turning to a more NS orientation over the Apennines foreland (Baccheschi et al., 2007; Civello & Margheriti, 2004; Lucente et al., 2006; Lucente & Margheriti, 2008; Margheriti et al., 2003; Petrescu et al., 2020; Plomerová et al., 2006; Salimbeni et al., 2013) (Figure 1b). The trench‐parallel anisotropy appears to be in contrast with the trench‐perpendicular P‐wave fast directions found by Hua et al. (2017), in correspondence of the isotropic high‐velocity anomaly imaged beneath the Northern Apennines as described in Section 1.2. Moving westward over the Tyrrhenian basin, SKS delay times increase and the fast azimuths, parallel to the stretching direction of the back‐arc basin, have been related to back‐arc extension (Faccenna et al., 2014; Jolivet et al., 2009; Lucente et al., 2006). More in general, EW‐oriented fast directions running parallel to the opening trajectory of the Liguro‐Provençal and Tyrrhenian basins are observed in the Central Mediterranean by both SKS and Rayleigh waves (Zhu & Tromp, 2013). Beneath the central Apennines, instead, smaller delay times have been interpreted as being due to a larger vertical component of shear and related steeply oriented fast axes (Lucente & Margheriti, 2008). The presence of trench‐perpendicular SKS fast azimuths has been related to either subduction windows or to toroidal mantle flow patterns at the edge of slabs. An example of the latter feature can be found in the Sicily channel where SKS fast axes turn to NS and then EW in the Tyrrhenian Sea (Baccheschi et al., 2007; Civello & Margheriti, 2004).

## 2 Methods

In this section, we describe the numerical methods used to generate subduction‐induced mantle flow patterns and to calculate strain‐induced mantle fabrics and synthetic seismic anisotropy.

### 2.1 Mechanical Numerical Modeling

3D petrological‐thermo‐mechanical models of subduction have been carried out with I3MG that is based on the finite difference method (FDM) combined with a marker‐in‐cell (MIC) technique (Gerya, 2019). The physical properties are defined on and advected by Lagrangian markers. A staggered Eulerian grid is defined to solve the equations of conservation of mass (Equation 1), momentum (Equation 2), and energy (Equation 3):

(1) $$\begin{array}{ccc} \nabla \cdot \vec{u}=0 & & \end{array}$$

(2) $$\begin{array}{ccc} -\nabla P+\nabla \cdot \tau =-\rho \vec{g} & & \end{array}$$

(3) $$\begin{array}{ccc} \rho C_{p}\frac{DT}{Dt}=-\nabla \cdot \vec{q}+H & & \end{array}$$

where D/Dt is the material time derivative and $\vec{u},\;P,\;\tau ,\;\rho ,\;C_{p},\;\vec{g},\;T,\;\vec{q}$ are the velocity, pressure, deviatoric stress, density, heat capacity, gravitational acceleration $\left(g_{x}=g_{z}=0,\;g_{y}=9.81\;ms^{-2}\right)$, temperature, and heat flux, respectively. The heat source term H accounts for radiogenic, adiabatic, and shear heating. The stable mineralogy and physical properties for the mantle used in our models are those computed with PERPLE_X (Connolly, 2005) and tested by Mishin et al. (2008) for a pyrolytic mantle composition. One of the main effect of taking thermodynamic databases into account is the incorporation of solid‐state phase transitions, with uncertainties on mineral thermodynamic properties that increase with depth. These transitions are associated with significant changes in mantle density and seismic wave speeds (Turcotte & Schubert, 2014), and affect the dynamics of mantle convection due to (a) density changes and (b) latent heating (Christensen & Yuen, 1985; Richter, 1973; Schubert et al., 1975; Tackley, 1993; Zhong & Gurnis, 1994). In our numerical models, the only major phase transition is the olivine‐spinel occurring at about 410 km depth (the spinel‐perovskite reaction at 670 km depth does not affect at all the model evolution that confined in the upper mantle and transition zone). Although the slab has a more depleted composition, it has been demonstrated that the density anomalies of a harzburgitic mantle are similar to those of a pyrolitic composition (Faccenda & Dal Zilio, 2017). This confirms what was found by the study of Nakagawa et al. (2009), which showed that even at global scale when using phase assemblages with different major oxides abundance and computed with the same software (PERPLE_X) the mantle convection patterns do not change substantially.

The numerical domain is a cartesian box defined by the (x−y−z) coordinates (2600 × 700 × 1800 km) discretized with (261 × 101 × 181 nodes), with y being the vertical direction. Velocity boundary conditions are free slip everywhere. We focus on modeling self‐consistent subduction driven by internal buoyancy forces (no kinematic conditions are prescribed). A constant temperature of 273K is applied at the top boundary, while a constant incoming heat flux of $2\;mW/m^{2}$ is imposed at the bottom boundary. The side boundaries are insulating.

The mantle mechanical behavior is modeled using a visco‐plastic rheology based on deformation invariants (Ranalli, 1995). The effective viscosity is given by the harmonic average of the combined dislocation, diffusion, and Peierls creep mechanisms (parameters and physical meaning are defined in Table 1):

(4) $$\eta_{ductile}={\left(\frac{1}{\eta_{disl}}+\frac{1}{\eta_{diff}}+\frac{1}{\eta_{peierls}}\right)}^{-1}$$

where the dislocation and diffusion creep are given by (Karato & Wu, 1993):

(5) $$\dot{\varepsilon }=A{\left(\frac{\sigma }{\mu }\right)}^{n}{\left(\frac{b}{d}\right)}^{m}exp\left(-\frac{E+PV}{RT}\right)$$

(6) $$\eta =\frac{\sigma }{2\dot{\varepsilon }}$$

**Table 1:** Physical Properties of Rocks Used in This Study

Property	Symbol	Value	Unit
Diffusion creep (Karato & Wu, 1993)			
Pre‐exponential factor	A	$8.7\cdot 10^{15}$	$s^{-1}$
Activation energy	E	300	$kJmol^{-1}$
Activation volume	V	4.5	$cm^{3}mol^{-1}$
Stress exponent	n	1	–
Grain‐size exponent	m	2.5	–
Burger vector	b	0.5	nm
Grain size	d	1	mm
Dislocation creep (Karato & Wu, 1993)			
Pre‐exponential factor	A	$3.5\cdot 10^{22}$	$s^{-1}$
Activation energy	E	540	$kJmol^{-1}$
Activation volume	V	17	$cm^{3}mol^{-1}$
Stress exponent	n	3.5	–
Grain‐size exponent	m	0	–
Peierls creep (Katayama & Karato, 2008)			
Pre‐exponential factor	A	$10^{7.8}$	$Pa^{2}s$
Activation energy	E	532	$kJmol^{-1}$
Activation volume	V	12	$cm^{3}mol^{-1}$
Peierls stress	$\sigma_{Peierls}$	9.1	GPa
Exponent	p, q	1,2	–, –

*Note*. $R=8.313\;Jmol^{-1}K^{-1}$ is the gas constant, while μ = 80 GPa is the shear modulus.

At elevated stresses (0.1GPa) typical of low‐T conditions creep is accommodated via the Peierls mechanism as (Katayama & Karato, 2008):

(7) $$\eta_{peierls}=0.5A\sigma_{II}^{\prime -1}exp\left\{\frac{E+PV}{RT}{\left[1-{\left(\frac{\sigma_{II}^{\prime }}{\sigma_{Peierls}}\right)}^{p}\right]}^{q}\right\}.$$

A pseudo‐plastic viscosity is computed as:

(8) $$\eta_{pl}=\frac{\tau_{y}}{2{\dot{\varepsilon }}_{II}}$$

Finally, the effective viscosity is given by:

(9) $$\eta_{eff}=min\left(\eta_{ductile},\eta_{pl}\right)$$

The plastic strength $\tau_{y}$ is determined with a plastic Drucker‐Prager criterion (Ranalli, 1995):

(10) $$\tau_{y}=C_{DP}+\mu P$$

where $C_{DP}=C\cos\phi =1MPa$ is the cohesion, μ=sinϕ is the friction coefficient, and ϕ is the friction angle. To model strain‐induced brittle weakening, the initial friction coefficient is linearly decreased upon reaching a final lower value when the accumulated plastic strain is 0.1. The lower and upper cutoff of the viscosity are set to $10^{18}$ and $10^{24}\;Pa\;s$.

The geometry of the Central Mediterranean was drawn according to the paleogeographic and tectonic reconstructions at ∼30 Ma proposed by Lucente and Speranza (2001), Lucente et al. (2006), Faccenna et al. (2014), and van Hinsbergen et al. (2014). The 3D initial temperature and compositional (crust vs. mantle) fields were created with the MATLAB toolbox geomIO (Bauville & Baumann, 2019) and then imported on I3MG to define the initial setup of the petrological‐thermo‐mechanical model.

**Figure 2:**
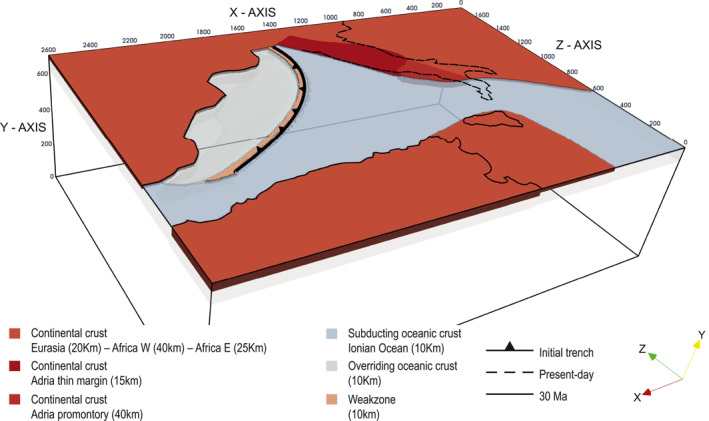
Initial model setup for the reference Model CM. It consists of a subducting oceanic plate (Ionian Ocean; light blue), two lateral continental blocks (Adria and Africa; orange), an overriding plate (Iberia and Europe: orange; thin upper plate: light gray) drawn according to paleogeographic reconstructions at 30 Ma proposed by Faccenna et al. (2014) and van Hinsbergen et al. (2014). The Adria plate is characterized by the presence of a stiffer continental promontory (dark orange) and a thin continental margin (red) as proposed by Lucente and Speranza (2001) and Lucente et al. (2006). A wide slab is located from Gibraltar to Corsica extending down to 300 km. The solid black line indicates the coastlines at 30 Ma (van Hinsbergen et al., 2014), while the dashed black line the present‐day coastlines of peninsular Italy.

The initial numerical model (Figure 2) consists of a subducting oceanic plate that represents the Ionian Ocean, two lateral continental blocks corresponding to the Adria and Africa plates, and an overriding plate that corresponds to the Iberia and European plates. To initiate subduction self‐consistently, (a) a wide slab is located from Gibraltar to Corsica dipping 40° and extending down to 300 km in the upper mantle, (b) a rheologically weak zone has been inserted on the slab top surface to lubricate the initial contact between the overriding and the subducting plates (constant viscosity of $10^{18}\;Pa\;s$ and constant density of $3200\;kg/m^{3}$), and (c) the overriding plate in between the trench and the European plate is composed of a young lithospheric portion that offers little resistance to slab roll‐back and trench retreat. The 300 km long slab is needed to trigger slab roll‐back self‐consistently, and it might be representative of a more recent stage of the Central Mediterranean history rather than the 30 Ma assumed here. In some models, the Adria plate structure, located in the current Apenninic area, is characterized by the presence of a stiffer continental promontory in its central portion and of a thin continental lithosphere in the Umbria‐Marche region (Calcagnile & Panza, 1980; Geiss, 1987; Lucente & Speranza, 2001; Lucente et al., 2006; Maino et al., 2013; Miller & Piana Agostinetti, 2012; Panza et al., 2003). The African plate consists of a slightly thinner margin on its eastern side toward the Ionian ocean (Arab et al., 2020). The initial lithosphere temperature distribution has been determined according to the half‐space cooling equation (Turcotte & Schubert, 2014), while the underlying asthenosphere is characterized by a constant adiabatic temperature gradient of $0.5\;Kkm^{-1}$. The continental plates (Africa, Africa eastern margin, Iberia, Adria, and Adria promontory) are set to be 150 Myr, the western‐northern portion of Adria is 90 Myr old to replicate a thin continental lithosphere, the young portion of the upper plate is 1 Myr to facilitate the trench retreat (we justify the young age by assuming a well‐developed continental rifting system North of the Balearics and Corsica‐Sardinia block). The subducting oceanic plate is 80 Myr old (which is representative of the thermal structure of the Piemonte‐Ligurian oceanic lithosphere 30 Ma ago; van Hinsbergen et al., 2014), while the slab in the mantle has an age of 70 Myr to model partial heating up by surrounding mantle. The density is calculated using the thermodynamic databases except in some models where for the crust of the continental promontory we use a constant value of $2700\;kg/m^{3}$. Otherwise, the continental crust density is computed as being that of the mantle minus $400\;kg/m^{3}$, except for the Adria thin margin where we subtract $200\;kg/m^{3}$ to model a less buoyant continental lithosphere. A detailed description of the crust and lithospheric mantle thicknesses together with other information for each plate can be found in Table 2.

**Table 2:** Rheological Parameters, Effective Thickness, and Age of the Different Plates for the Reference Model CM

Plate name	Effective thickness		Friction coefficient (–)		Density ($kg/m^{3}$)
	(km)				
MODEL CM					
*Lithosphere*	*Crust*	*Mantle*	*Initial*	*Final*	
Eurasia (150 Myr)	20		0.020	0.005	$\rho_{mantle}-400$
		60	0.600	0.400	$\rho_{mantle}$
Africa W (150 Myr)	40		0.020	0.005	$\rho_{mantle}-400$
		60	0.600	0.400	$\rho_{mantle}$
Africa E (150 Myr)	25		0.020	0.005	$\rho_{mantle}-400$
		65	0.600	0.400	$\rho_{mantle}$
Adria promontory (150 Myr)	40		0.600	0.400	2700
		50	0.600	0.400	$\rho_{mantle}$
Adria thin margin (90 Myr)	15		0.020	0.005	$\rho_{mantle}-200$
		70	0.600	0.400	$\rho_{mantle}$
Ionian Ocean (90 Myr)	10		0.020	0.005	$\rho_{mantle}$
		80	0.600	0.400	$\rho_{mantle}$
Upper plate (1 Myr)	10		0.010	0.010	$\rho_{mantle}$
		80	0.600	0.400	$\rho_{mantle}$
Weakzone (70 Myr)	20	–	–	–	3200
MODEL A					
Adria thin margin is absent					
MODEL B					
Adria promontory is absent					
MODEL C‐F1‐F2					
Adria promontory and Adria thin margin are absent					
MODEL D1					
Adria Promontory crust	”	”	”	”	$\rho_{mantle}-500$
MODEL D2					
Adria Promontory crust	”	”	”	”	$\rho_{mantle}-400$
MODEL D3					
Adria Promontory crust	”	”	”	”	$\rho_{mantle}-300$
MODEL D4					
Adria thin margin crust	”	”	”	”	$\rho_{mantle}-400$
MODEL E					
Adria Promontory crust	”	”	0.020	0.005	”

*Note*. For all the other models, we only indicate the differences with respect to model CM. $\rho_{mantle}$ is the mantle density from the thermodynamic databases. The viscosity of the weakzone is constant ($10^{18}\;Pas$). The employed low friction coefficients for crustal material ensure lubrication at the plates contact and one‐sided, terrestrial‐like subduction geometry (Gerya et al., 2008).

### 2.2 Strain‐Induced LPO

The development of crystal aggregates LPO in the upper mantle depends on several deformation mechanisms, including plastic deformation, dynamic recrystallization (by sub‐grain rotation and grain‐boundary migration), and grain‐boundary sliding (Kaminski et al., 2004). Here, we use a modified version of D‐REX (Kaminski et al., 2004), that incorporates these deformation mechanisms to compute the LPO and accounts for the non‐steady‐state evolution of geodynamic systems (Faccenda & Capitanio, 2013).

Lagrangian particles representing upper and transition zone mantle mineral aggregates are regularly distributed throughout the computational domain (25 km reciprocal distance along the three directions, for a total of 209,664 aggregates). Each particle consists of 1,024 randomly oriented crystals forming an initially isotropic upper mantle with harzburgitic composition (70% olivine and 30% orthopyroxene modal abundance) and transition zone mantle with a more fertile, pyrolitic composition (60% spinel and 40% majoritic garnet) (Faccenda, 2014). These particles are then advected by means of the Eulerian velocity field obtained by the macro‐flow modeling. At each time step, the orientations of these crystals change in response to gradients in the velocity field, generating LPO. We only compute the strain‐induced fabrics in the upper mantle (from the Moho to the 410 km discontinuity) as SKS splitting parameters are mostly sensitive to this mantle layer (Sieminski et al., 2008). The upper mantle fabrics are reset and transformed to isotropic transition zone mantle aggregates upon crossing the Olivine‐Spinel phase transition, and vice versa. Crystal aggregates of the transition zone are always assumed to be seismically isotropic (i.e., random crystal orientation). We use the same dimensionless crystallographic parameters as in Rappisi and Faccenda (2019) with the nucleation rate $\lambda^{*}=5$, the grain‐boundary‐mobility $M^{*}=1$ and the threshold volume fraction $\chi^{*}=0.9$. When compared to previously published parameters calibrated with laboratory experiments (e.g., Boneh et al., 2015; Faccenda & Capitanio, 2013; Kaminski et al., 2004), the low $M^{*}$ and high $\chi^{*}$ yield weaker fabrics and smaller amounts of seismic anisotropy that are more consistent with seismological observations.

### 2.3 SKS Splitting

The synthetic SKS splitting is calculated using routines included in the software package FSTRACK (Becker, 2006). The code first obtains a pulse seismogram via inverse Fourier transform using the method of Kennett (2009) with anisotropic extensions (Booth & Crampin, 1983; Chapman & Shearer, 1989) by computing the harmonic response of a horizontal layer stack to an incident plane wave (5° for typical SKS arrivals) over a range of frequencies (0–25 Hz). Synthetic seismograms in the SKS band (3.3–10 s) are then constructed applying band‐pass filters from 0.1 to 0.3 Hz. Successively, the splitting is determined with the cross‐correlation method of Menke and Levin (2003). A 2D grid of virtual seismic stations regularly spaced is constructed in the model. After recovering the elastic tensors from each aggregate's stiffness matrix, we build below each station and down to 400 km a vertical stack of horizontal layers (minimum thickness of 25 km) where the elastic tensor of each layer is radially averaged within a distance of 50 km. The SKS splitting parameters of each seismic station are obtained by averaging all the fast azimuths and delay times measured by rotating the vertical stack of elastic tensors by 5° intervals around the *y*‐axis.

### 2.4 P‐Wave Anisotropy and S‐Wave Azimuthal Anisotropy

P‐wave anisotropy is computed for each crystal aggregate as $Vp_{anis}\left(\%\right)=\left(Vp_{max}-Vp_{min}\right)/\left(Vp_{max}+Vp_{min}\right)*200$, where $Vp_{max}$ and $Vp_{min}$ are the max. and min. P‐wave velocities evaluated for different incidence angles and azimuths. Another precious source of information is represented by the azimuthal anisotropy of Rayleigh waves, which however has a low lateral resolution. To roughly simulate this effect, we have interpolated the elastic tensors of crystal aggregates to a grid with node spacing of 100 × 25 × 100km. The fast azimuth ϕ and magnitude G of the S‐wave azimuthal anisotropy are then computed as, respectively:

(11) $$\begin{array}{ccc} \phi =0.5\cdot \tan^{-1}\left(G_{s}/G_{c}\right) & & \end{array}$$

(12) $$\begin{array}{ccc} G=\sqrt{G_{c}^{2}+G_{s}^{2}} & & \end{array}$$

where $G_{c}=\left(C_{55}-C_{44}\right)/2$, $G_{s}=C_{45}$ and $C_{55},C_{44},C_{45}$ are the elastic tensor moduli.

## 3 Results

### 3.1 Reference Model CM

In this section, we describe in detail the tectonic evolution of the Reference Model CM. This model, among the several ones that have been tested as a function of the rheological parameters and of the plates structural/geometrical characteristics, is capable of reproducing the main geological and geophysical observations of the Central Mediterranean given the intrinsic model limitations. The influence of the imposed structural heterogeneities and rheological parameters are discussed in the next section.

**Figure 3:**
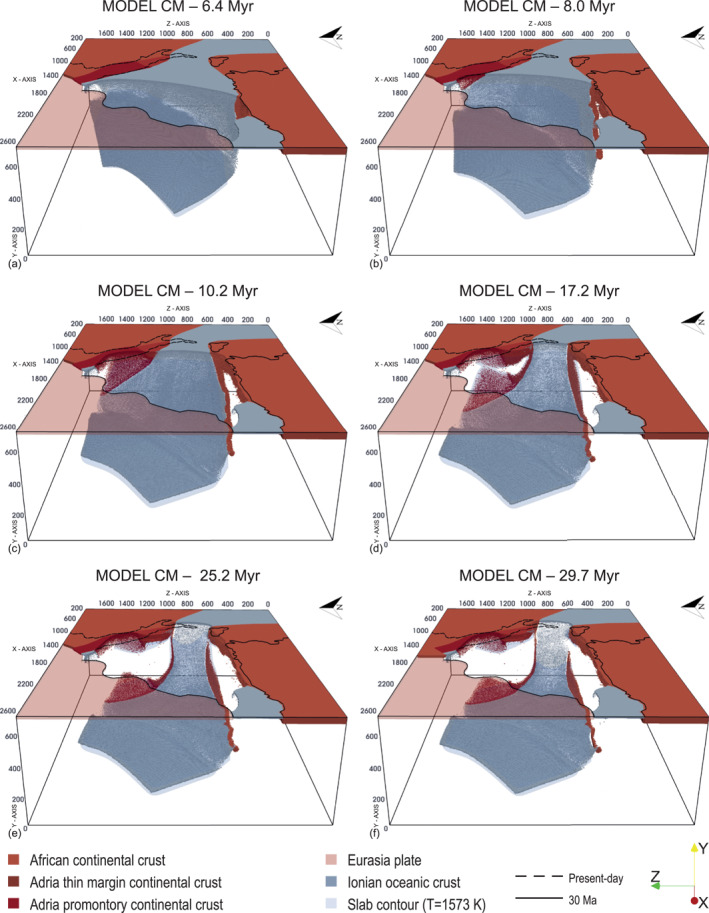
Snapshots of the Reference Model CM evolution. In light gray, the subducted slab (contour at *T* = 1,573K) below ∼120 km depth. The Eurasian plate was opacified in the 1,600 < *x* < 2,600 km range for a better visualization of the subducted slab. The solid black line indicates the coastlines at 30 Ma (van Hinsbergen et al., 2014), while the dashed black line indicates the present‐day coastlines of peninsular Italy.

The Reference Model CM is characterized by the presence of structural heterogeneities in the Adria continental plate, that is, a thinned margin in its northern portion and a stiffer promontory in its center as proposed by Lucente and Speranza (2001) and Lucente et al. (2006) (Figure 2). In this model, the subduction, driven solely by the slab negative buoyancy, initiates through progressive bending and roll‐back of the oceanic plate, and causes a homogeneous stretching of the overriding lithosphere (Figure 3, Movie S1). The Ionian slab and trench rapidly migrate south‐eastward with episodes of slab lateral tearing, segmentation, and break‐off when a continental margin enters the trench.

Specifically, the western part of the trench reaches the African continent in ∼4.5 Myr. When the trench impacts with the African margin, part of the continental lithosphere subducts and reaches the depth of ∼150 km, and at ∼6.4 Myr slab tearing occurs at the transition between the oceanic lithosphere and the more buoyant continental lithosphere (Figure 3a). Lithosphere tearing subsequently propagates eastward along the African passive margin, favoring slab rollback toward the east.

At ∼7–8Myr (Figure 3b) the north‐eastern portion of the trench progressively collides with the thin north‐western margin of the Adria plate and the subsequent continental crust subduction induces (a) along‐trench buoyancy variations resulting in a progressive curvature of the trench and (b) tearing of the slab north‐eastern edge along the oceanic‐continental lithosphere boundary (Figure 3c). After ∼10 Myr, the trench reaches Central Adria with partial subduction of the stiffer continental promontory. At the same time, the subducting slab has already reached the lower boundary of the model and stagnates horizontally in the mantle transition zone.

Between ∼15 and ∼16 Myr, slab break‐off occurs beneath the Adria promontory. The rupture rapidly propagates laterally, allowing, in only a few million years, the formation of a wide slab window that breaks the single arc geometry and creating two separated arcs (Figure 3d). The slab northern segment retreats eastward until it breaks at a depth between 150 and 200 km (∼18 Myr), while the remaining Ionian slab becomes narrower and continues to migrate south‐eastward.

After ∼25–30 Myr (Figures 3e and 3f), slab remnants are found in model areas corresponding to the present‐day northern Apennines and southern Tyrrhenian sea. The northern slab segment is hanging down to ∼150 km depth and extends deeper from ∼400 km down to about ∼660 km depth. The southern slab segment instead extends continuously from the surface down to the mantle transition zone although incipient detachment is observed in the eastern African margin.

### 3.2 Comparison Between the Reference Model CM and Other Models

Here, we want to evaluate the influence on trench shape and on the occurrence and timing of slab tears (e.g., Mason et al., 2010) of the structural heterogeneities within the Adria plate. We explore a wide range of models (Table 2) where, with respect to the Reference Model CM, we varied the geometry of the Adria plate and the buoyancy and stiffness of the Adria promontory and Adria thin margin crust. Unfortunately, the past extent of the Adria plate and the geometry of its passive margins are quite uncertain, mostly because the latter are now subducted. As a consequence, in the literature there appears to be consensus on the Mid‐to‐Late Cenozoic kinematic evolution of the Ionian subduction margin, while few studies have attempted to reconstruct the distribution of oceanic and continental portions of the Adria plate, and thus the geometry of its margins. For this reason, here we have tested different potential margin geometries and plate configurations (homogeneous vs. heterogeneous plate, straight vs. curved passive margin), partly following those suggested in the literature (Faccenna et al., 2014; Lucente & Speranza, 2001; van Hinsbergen et al., 2014), and partly hypothesizing some potential geometries like those depicted in Figure 6.

As expected, the initial subduction dynamics of all models are similar to that of Model CM: the initial slab sinks through the mantle and retreats to the south‐east. When the trench impacts with the African margin, lateral slab tearing favors subsequent eastward rollback. Important differences among models occur when the trench reaches the Adria continental margin.

**Figure 4:**
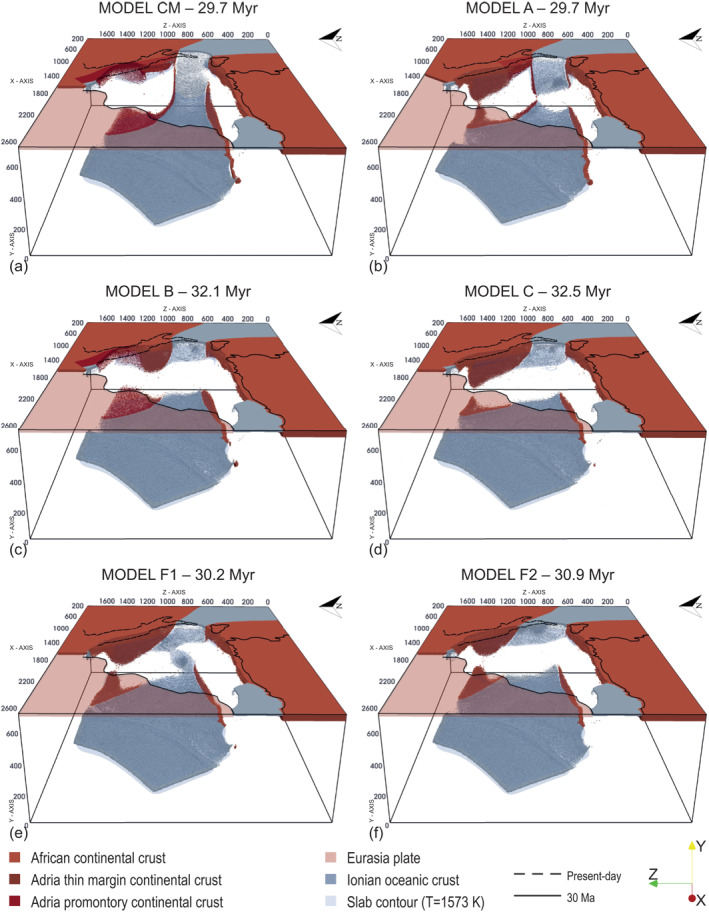
Snapshot at ∼30 Myr of: (a) Model CM, (b) Model A, (c) Model B, (d) Model C, (e) Model F1, and (f) Model F2. Color‐coding and coastlines as in Figure 3.

**Figure 5:**
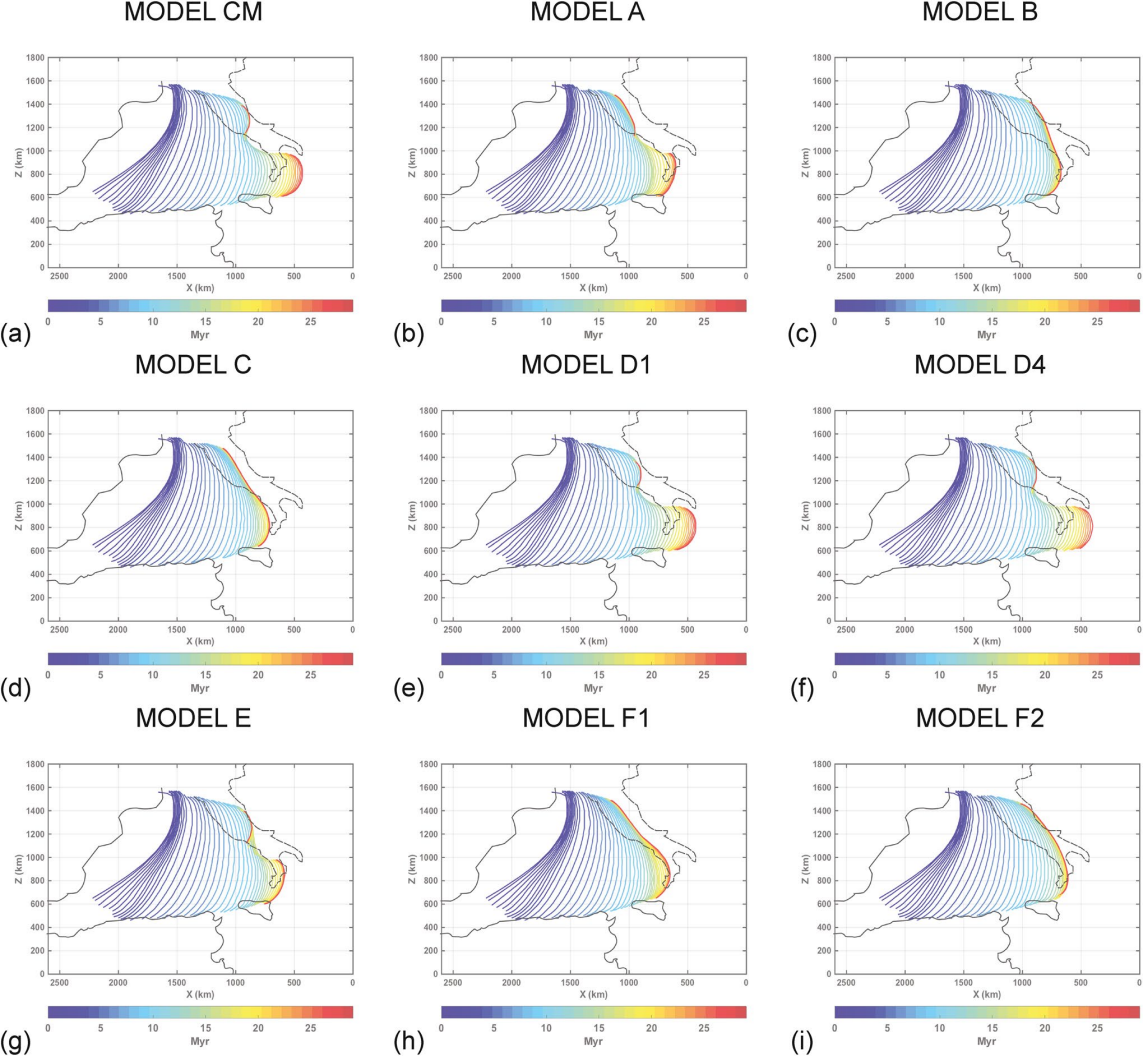
Top view of the trench position evolution up to 30 Myr for: (a) Model CM, (b) Model A, (c) Model B, (d) Model C, (e) Model D1, (f) Model D4, (g) Model E, (h) Model F1, and (i) Model F2. The color bar indicates the time in million years. In models CM, B, D1, D4, E, and F2, the northern section of the trench retreats till the Adriatic Sea in ∼30 Myr. The retreat of the Ionian slab till the present‐day position in the Calabro‐Peloritan region is reproduced in models CM, D1, and D4. Coastlines as in Figure 2.

When the thin continental margin in the northern Adria plate is absent (Model A), the eastward migration of the northern Ionian trench is hindered and slab tearing below the Adria promontory occurs ∼3 Myr later than in the Model CM (∼19 Myr). Over the next few millions of years, the tear spreads laterally forming a wide slab window (Figure S1b) and leading to complete slab detachment at ∼30 Myr (Figure 4b) with slab remnants down to ∼350 km depth. This results in a lower amount of retreat of the northern Ionian trench, whose final shape appears linear and not arcuate as in Model CM (Figures 5a, 5b, S2b and S2c).

When the Adria promontory is not included (Model B), the central slab window and the consequent double arc geometry are not generated. At ∼20 Myr the northern part of the trench is curved and has reached the same position as Model CM (Figures 5a and 5c). The Ionian slab totally breaks off between ∼24 and ∼25 Myr (Figure S1c), resulting in a smaller amount of trench retreat to the south when compared to Model CM (Figures 5a, 5c, S2b and S2d). At ∼30 Myr, we find remains of hanging subducted lithosphere only in the central part of the Adria plate (Figure 4c).

When both the stiff continental promontory and the thin continental margin are removed (Model C), a tear occurs at ∼21 Myr in the slab central portion and again quickly spreads toward its edges. At ∼23 Myr, the southern edge of the slab breaks at a depth between 150 and 200 km (Figure S1d), while the remaining northern slab breaks at a depth of ∼300 km at ∼30 Myr (Figure 4d). The final position of the trench is equal to Model A in the northern part and equal to Model B in the southern part (Figures 5b–5d and S2c–S2e).

The continental crust is much more buoyant with a density of ∼2,700–3,000 $kg/m^{3}$, compared to 3,300 $kg/m^{3}$ for the mantle. Here, we further test the influence of the density of the promontory continental crust in models D1, D2, and D3, which is 500, 400, and $300\;kg/m^{3}$, respectively, lighter than the mantle. This density is higher than that in Model CM where it was set constant and equal to 2,700 kg/m3. The models evolution remains very similar to Model CM, and a wide slab window develops leading to the formation of two separate arcs after the subduction of the denser continental promontory. At ∼30 Myr the shape and position of the two trenches of these three models are very similar to Model CM (Figures 5a and 5e). An analogous result was obtained for Model D4, where a more buoyant Adria thin continental margin is tested by decreasing the continental crust by $200\;kg/m^{3}$ (i.e., $\rho_{crust}=\rho_{mantle}-400\;kg/m^{3}$) relative to that of Model CM (Figures 5a and 5f). In general, the influence of continental crust density of different Adria plate domains is minor and exerts negligible effects on the final model evolution.

In Model E, we have instead inserted a less rigid promontory, whose initial and final friction coefficients are set equal to those of the Adria plate (0.020 and 0.005). Although the overall evolution of the subduction is similar to that of the Model CM, the timing of slab break‐off is different. At ∼25 Myr, total slab detachment has already occurred in both the northern and southern slabs, that is, at least 5 million years earlier than the Model CM. This causes a minor retreat of the southern trench (Figures 5a, 5g, S2b and S2f).

**Figure 6:**
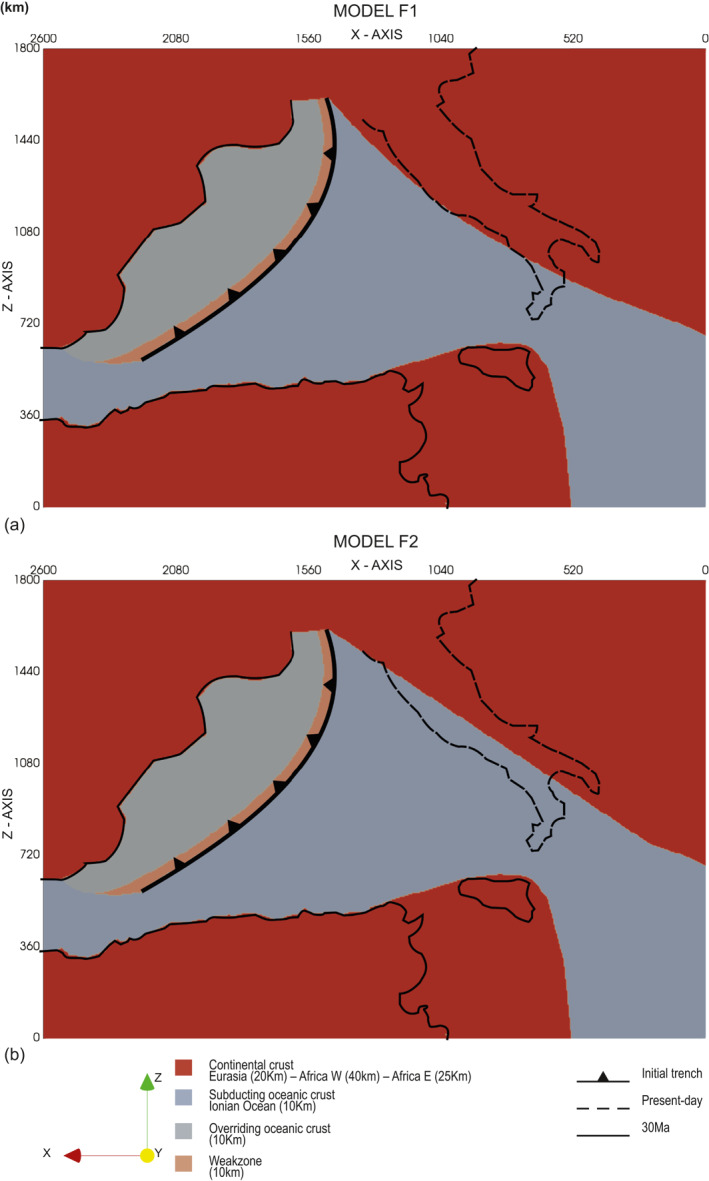
Top view of the initial model setup for the models with a homogeneous Adria plate (without structural heterogeneities): (a) Model F1 and (b) Model F2). Coastlines as in Figure 2.

Finally, we tested two models (models F1 and F2) with a homogeneous Adria plate (without lateral heterogeneities). In these models, the initial geometry of the Adria plate is simpler than in Model CM (Figure 6). Model F1 shows a westward convex geometry of the Adria plate western margin (Figure 6a), which is instead straight in Model F2 (Figure 6b). In Model F1, the evolution of the Ionian slab in the mantle is well reproduced (Movie S2), with the formation of the wide slab window under the central part of Adria (Figure S1e). At ∼30 Myr the northern slab is broken at a depth of about 200 km while the southern slab is still continuous (Figure 4e). Compared to the Model CM, however, the retreat of the two trenches is smaller. Furthermore, the trench arcuate shape observed in Model CM is not recreated (Figures 5a, 5h, S2b and S2g). The evolution of Model F2 is instead similar to that of Model B. When the Adria plate passive margin enters the trench, the slab breaks off completely without developing the slab window and the two separate arcs (Figure S1f). At ∼30 Myr, we find remains of subducted lithosphere only in the central part of the Adria plate (Figure 4f). Also, in this case, the final shape of the trench is linear (Figures 5i and S2h).

### 3.3 Upper Mantle Flow, LPO, and Synthetic Seismic Anisotropy

The mantle flow induced by slab subduction for Model CM is characterized by the presence of poloidal and toroidal components (Movie S1). The initial slab subduction is associated with a dominant poloidal asthenospheric flow and mantle upwelling in the mantle wedge. At the same time, slab rollback is accommodated by return flow from the sub‐slab region toward the mantle wedge around the slab edges and, at later stages, through the newly formed slab window.

**Figure 7:**
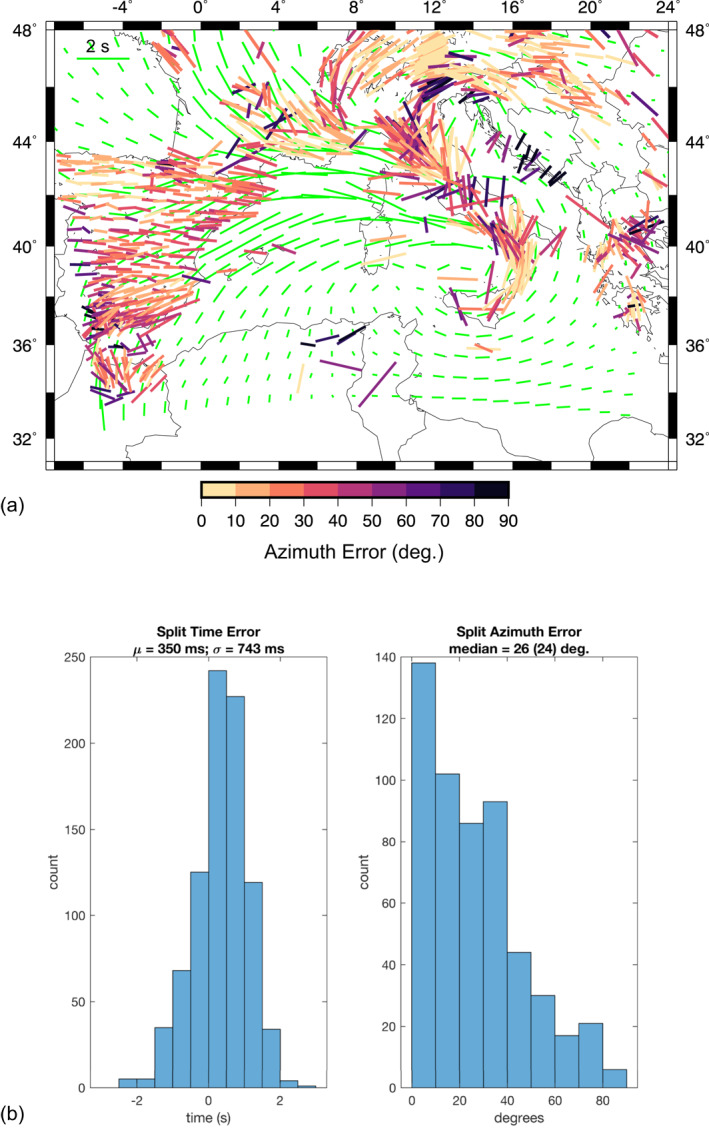
(a) SKS‐splitting measurements in the Central‐Western Mediterranean (Becker et al., 2012) color‐coded by the error in the orientation compared with synthetic SKS splitting measurements for Model CM at ∼20 My (green bars). The EW green bar in the upper left corner indicates 2 s. (b) Split time error and split azimuth error histograms.

**Figure 8:**
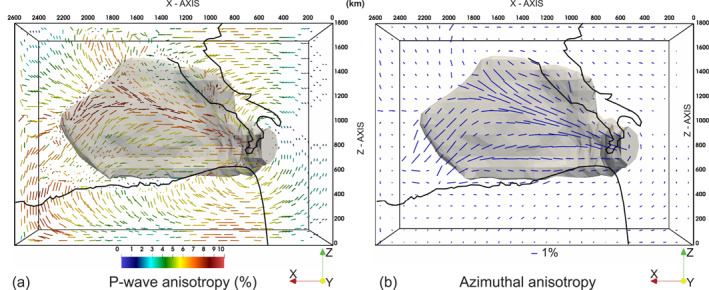
Top‐view of the Reference Model CM at ∼30 Myr showing (a) $Vp_{anis}$ for selected aggregates at a depth of 150 km, with the length and color of each bar proportional to the amount of anisotropy and the direction indicating the direction of $Vp_{max}$; (b) The S‐wave azimuthal anisotropy at a depth of 140 km with the orientation and length of the blue bars representing the fast azimuth Φ and magnitude G of azimuthal anisotropy, respectively. The EW blue bar outside the domain indicates 1% of azimuthal anisotropy. The gray surface shows the material with P‐wave (left) and S‐wave (right) isotropic velocity anomaly 1% with respect to the depth averages velocities below 140 km depth, and corresponds to the Ionian slab. The coastlines of Africa at 30 Ma and the present‐day coastlines of peninsular Italy are indicated in black for reference.

**Figure 9:**
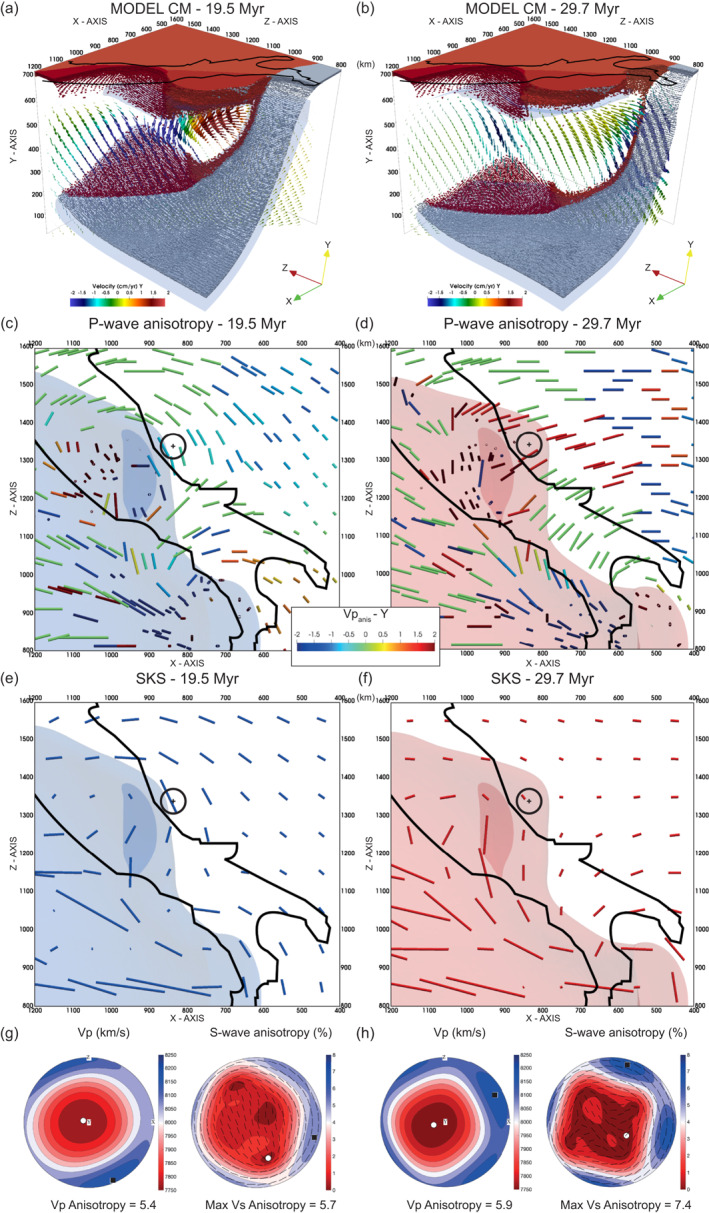
Compositional field and seismic anisotropy in the reference Model CM at ∼20 Myr (left column) and ∼30 Myr (right column). First row is the compositional field cropped around the region corresponding to the Apenninic belt with a vertical slice of the velocity field crossing the Apennines. The arrows length is proportional to the velocity magnitude, and are color‐coded according to the velocity vertical (*Y*) component. Second row is the P‐wave anisotropy at 137.5 km depth. The bars length is proportional to the $Vp_{anis}$ magnitude, and are color‐coded according to the $Vp_{anis}$ vertical (*Y*) component. Third row is the SKS splitting. The transparent blue and red volumes denote the lithosphere deeper (lighter) or shallower (darker) than 200 km depth. Fourth row shows the pole projections P‐wave velocity (m/s) and $Vs_{anis}=\left(Vs_{1}-Vs_{2}\right)/\left(Vs_{1}+Vs_{2}\right)*200$ as a function of the propagation direction for a mantle crystal aggregates representative of the northern Apennines area (X=837.5km, Y=137.5km, and Z=1337.5km; shown by the circled cross in panels (c–f)). The bars in the $Vs_{anis}$ plot indicate the polarization direction of the $Vs_{1}$ component. The black square and white circle indicate max. and min. values, respectively.

The evolution of the subduction margin produces strong upper mantle fabrics in the area surrounding the retreating slab to a depth of 410 km and considerable amount of seismic anisotropy. In the supra‐slab upper mantle, the general trend of the fast azimuths is trench‐perpendicular, while the sub‐slab mantle is characterized by trench‐parallel extension and anisotropy (Faccenda & Capitanio, 2012, 2013) (Figures 7, 8 and S2). In areas characterized by slab break‐off/lateral tearing and vertical flow such as around the modeled Northern Apennines and Calabrian arc, the shear wave anisotropy is relatively small due to the dipping fast axes of olivine crystals at shallow mantle depths (to which SKS phases are mostly sensitive). Synthetic SKS splitting measurements (Figures 7 and S2b) indicate that the fast shear wave component orients perpendicular to the past trench positions in the back‐arc region. This results in a smoothly rotating direction pattern, with a dominance of NW directions in the north turning progressively toward EW. The back‐arc region is characterized by very high delay times (δt=2−3.4s), which reduce closer to the trench (δt<1.5s) and orient parallel to it in the fore arc region. Around the slab edges, the teleseismic fast shear wave component traces the underlying return flow (δt=1−2s) with a circular pattern surrounding the southern and the norther trenches. The P‐wave and Rayleigh wave fast azimuths are consistent with those of the SKS‐splitting data (Figure 8). Around the region corresponding to the northern Apennines there is significant change in P‐wave and SKS anisotropic patterns between ∼20 and ∼30 Myr (Figure 9). At earlier stages, strong and trench‐parallel P‐wave and SKS anisotropy results from the retreating western Adria margin (Figures 9c and 9e). At later stages, however, the trench‐parallel SKS fast azimuths are substantially reduced and the P‐wave fast directions at 130 km depth turn to trench‐perpendicular dipping toward the sinking and detached Ionian slab (Figures 9d and 9f). This is because the detached Ionian slab induces a downward and radially converging mass flux (Figures 9a and 9b) in the shallow upper mantle that progressively re‐orients the fast axis of the olivine crystals from ∼NW to ∼NE (Figures 9g and 9h; Vp pole figures). The change in fast axis dip from sub‐horizontal to SW dipping is responsible for the decrease in SKS splitting δt (Figures 9g and 9h; S‐wave anisotropy pole figures).

The mantle flow evolution of the other models (A, B, C, D1, D2, D3, D4, E, F1 [Movie S2], and F2) is similar to Model CM, where lithospheric deformation and asthenospheric flow are controlled by the slab roll‐back and poloidal and toroidal components of mantle circulation are both active. An important difference occurs in the models where a shallow slab window does not form (B, C, F1, and F2). In this case, the return flow entering the window is not established and a larger toroidal flow component around the edges of the wider slab is observed.

We also calculated the synthetic SKS splitting parameters for models A, B, C, E, F1, and F2 (Figures S2c–S2h). In the bark‐arc region, SKS splitting patterns of these models are quite similar to those of Model CM, with a fast shear wave component aligned trench‐perpendicular and showing an anticlockwise rotating pattern. Substantial differences are found in regions near the trench affected by the slab break‐off and lateral tearing. In the models A, B, C, E, F1, and F2, the clockwise pattern of the fast azimuths surrounding the southern trench is characterized by shorter delay times (δt<1s) due to a lower amount of trench retreat (Figures  S2c–S2h, 5b–5d, and 5g–5i). Conversely, in models A, C, F1, and F2 (Figures S2c, S2e, S2g, and S2h), the circular pattern surrounding the northern trench is well highlighted by the higher delay times (δt=1−1.5s). This reflects the strong toroidal flow establishing around the rectilinear slab present at shallow depths along the entire Adria plate. Furthermore, in models B, C, F1, and F2 (Figures S2d, S2e, S2g, and S2h), differences emerge in the Adria central portion where the fast axes orient parallel to the trench as the absence of the slab window does not produce any return flow through it.

## 4 Discussion

### 4.1 Data and Model Limitations

The direct application of numerical modeling to natural cases is affected by the modeling assumptions and limitations. For example, in our large‐scale geodynamic models, the relative position of the plates slightly differs from the present‐day one. This is because we used an initial configuration defined at ∼30 Ma according to the paleogeographic and tectonic reconstructions from Lucente and Speranza (2001), Lucente et al. (2006), Faccenna et al. (2014), and van Hinsbergen et al. (2014), and we have not applied a convergence rate between the plates (self‐consistent subduction). As such, the African continent never reaches its current position. It is worth noting though that we mainly focus on the subduction system and its effect on mantle flow and anisotropy patterns. An initial geometry defined at ∼30 Ma should not have a strong impact on mantle flow directions and splitting parameters since the slow Africa‐Europe plates convergence has not caused a drastic change in plates position over this time span.

The model domain is defined in Cartesian coordinates, which could cause discrepancies when comparing our synthetic observations with the real measurements (e.g., Figure 7). Furthermore, tectonic reconstructions show that at ∼30 Ma, an incipient slab (150 km), located from Gibraltar to Corsica, was already subducted in the upper mantle (Faccenna et al., 2014; van Hinsbergen et al., 2014). However, to trigger a “spontaneous” subduction system, the trench has been initially positioned further south with the slab extending to a depth of 300 km to model a more developed subduction and increase the slab negative buoyancy. This could cause a difference in rates of Ionian slab retreat at the model early stage when compared to those reported in the literature.

The Central Mediterranean dynamics has been likely affected by the presence of multiple subducting slabs rather than a single one as in our models. Numerical and analog models with multiple subductions demonstrated that the number and geometry of interacting slabs strongly affect the overall force balance, and that interactions through the mantle can exert a primary control on the geometry and kinematics of subduction (Holt et al., 2017, 2018; Jagoutz et al., 2015; Luth et al., 2013; Peral et al., 2020). In addition, it remains difficult if not impossible to evaluate the role of other structural heterogeneities (i.e., oceanic transform faults) and geometrical complexities that might have been potentially present within the subducted Ionian plate and eastern Adria margin and that have not been considered here.

It is important to point out that validation of geodynamic models through comparison of the seismological synthetics with observations is limited by (a) uncertainties concerning the seismological data sets and related to the fact that, for example, a good azimuthally coverage is needed to properly describe the back‐azimuthal dependency of the SKS splitting parameters, (b) lack of anisotropy data in oceanic regions and certain continental areas (i.e., Central Apennines), and (c) ignoring other sources of seismic anisotropy in our models. The latter include fossil LPO fabrics in the crust and mantle of the subducted and overriding lithosphere, and preferentially aligned compositional heterogeneities and fluid‐filled cracks. About crustal fabrics, we note that SKS and SKKS waves have a wide lateral sensitivity (the Fresnel zone is typically on the order to 100 km or more in the upper mantle), and the role of the few tens of km thick subducted continental crust on seismic anisotropy of long‐wavelength waves is likely minor. Little is known about the mantle fossil anisotropy which might be present in the Ionian‐Adria lithosphere, but we note that it would be quite a coincidence the situation where the orientation of the rotated (i.e., subducted) fabrics is consistent with the systematic along‐strike orientation of the SKS fast azimuths along the mountain belts with a very complex arcuate geometry. As an example, Petrescu et al. (2020) carried out Fresnel analysis of the SKS splitting data with trench‐parallel patterns along Alps and placed the source of anisotropy at asthenospheric depths. The anisotropy produced by fluid‐filled, 10 km thick crustal faults in the Apennines is typically small (<0.1 s in average; Baccheschi et al., 2016), while Faccenda et al. (2019) have demonstrated that extrinsic anisotropy due compositional heterogeneities is negligible. Hence, we believe that strain‐induced LPO fabrics in the recently deforming hot mantle should explain most of the observations in the area.

Modeling of strain‐induced LPO in upper mantle rocks has also some limitations related to the incomplete knowledge of the relative contribution of the different creep mechanisms as a function of the local P‐T conditions, and to the employed numerical approach (for a recent review of this topic, see Hansen et al., 2021).

Despite these limitations, we find a good correspondence between the modeled and observed surface and deep isotropic structures, and seismic anisotropy patterns. This might indicate that our numerical geodynamic models are able to capture the first order of the overall evolution and the current geological scenario of the Central Mediterranean. In particular, the models reproduce several episodes of slab lateral tearing and break‐off that have been proposed according to geological and seismological data.

### 4.2 Comparison With Seismic Tomography Models

A common feature of all of our synthetic models is a high‐velocity body (Figure 8) arranged horizontally over the 660 km discontinuity. This high‐velocity anomaly is a prominent feature in P‐wave tomographic models (Amato et al., 1993; Lucente et al., 1999; Piromallo & Morelli, 2003; Selvaggi & Chiarabba, 1995; Spakman et al., 1993; Spakman & Wortel, 2004; Van der Meer et al., 2018) and it has been interpreted as the Ionian slab lying and broadening at the base of the upper mantle.

The slab remnants found in the later stages of Model CM under the regions corresponding to the Northern Apennines and Calabrian arc are consistent with (a) the observed intermediate and deep seismicity (Chiarabba et al., 2005), and (b) the two pronounced high‐speed surface anomalies separated by a slow velocity anomaly (Central Apennines Slab Gap) detected by seismic tomography models. A high‐velocity anomaly is detected at 250 km and deeper below the entire Apenninic belt that can be related to subducted Ionian oceanic lithosphere. The portion of the slab under the northern Apennines extends down to 150 km depth and from ∼200 km down to about 660 km depth (Figures 3d and 9a). This is consistent with recent tomographic studies (El‐Sharkawy et al., 2020; Giacomuzzi et al., 2012; Spakman & Wortel, 2004) suggesting that the Northern Apennines slab reaches a depth of only 150–300 km. The vertical width of the slab gap increases with time, such that top portion of the detached slab is at 400 km depth at ∼30 Myr (Figures 3f, 4a and 9b). The northern section of the modeled trench retreats westward and already at ∼20 Myr resembles the arcuate shape of the present‐day northern Apennines extending from the western Po Plain to the Adriatic Sea. Toward the south, the modeled Ionian slab reaches its present‐day position in the Calabro‐Peloritan region again at ∼20 Myr (Figure 5a). According to Model CM (Figures 3f and 4a), the Calabrian slab is continuous from the surface up to a depth of 660 km beneath the northern and central Calabria but the detachment has already occurred beneath north eastern Sicily (El‐Sharkawy et al., 2020; Giacomuzzi et al., 2012; Neri et al., 2012; Presti et al., 2019; Scarfì et al., 2018). Thus, the modeled structures of Model CM at ∼20 Myr appear to be quite compatible with the present‐day surface and deeper configuration (Figure 9, left column). This is in agreement with the fact that our initial model geometry characterized by a deeper slab than what reconstructed at ∼30 Ma by Faccenna et al. (2014) and van Hinsbergen et al. (2014) is likely representative of a more a recent stage of the Ionian subduction.

A substantially similar evolution was obtained for the models A, D1, D2, D3, D4, E, and F1 (Figures 4b and 4e), although with a minor retreat of the trenches in the models A, E, and F1 (Figures 5b, 5g, 5h, S2c, S2f, and  S2g). This implies that continental crust thickness and strength, rather than its density contrast with respect to the mantle (models D1, D2, D3, and D4), play a major role in defining the modeled final configuration.

Results inconsistent with tomography observations were instead obtained for the models B, C, and F2, where the absence of the stiffer Adria promontory causes the complete break‐off of the Ionian slab (Figures 4c, 4d, and 4f). In Model B, we find hanging subducted lithosphere only below the central Apennines, which is in contrast with seismic tomographies supporting the presence of a slab gap (Figure 4c). In models C and F2, a hanging slab is found under the entire Apenninic chain (Figures 4d and 4f). Moreover, the absence of a thin continental margin in the northern Adria plate (models A, C, F1, and F2) hinders the western retreat of the northern trench and the formation of an arcuated trench similar to that observed along the Apenninic chain (Figures 5b, 5d, 5h, 5i, S2c, S2e, S2g, and S2h). It is important to mention that including a free surface condition near the top boundary could affect the final geometry of the model in the continental areas, as portions of the continental crust entering the trench could be thrusted over the foreland, shifting the trench more eastward (Faccenda et al., 2009).

In synthesis, our geodynamics experiments highlighted that the initial subduction dynamics of all models are similar and important differences in terms of rollback rates, trench shape, and occurrence and timing of slab tears occur in the final stages of their evolution when the Adria continental margin enters the trench. In particular, our results show that:

1. The presence of a thin continental lithosphere (models CM, B, D1, D2, D3, D4, and E) favors the curvature of the Northern Apennines trench and its retreat till the Adriatic Sea, which suggests that the arched shape of the northern Apennines is a consequence of deep processes occurring inside the Earth, that is, the lateral flexion of the subducting Adriatic plate (Lucente & Speranza, 2001);
2. The development of a slab window below the Central Apennines and double arcs geometry, as shown by seismic tomographies, occurs in the models where a stiffer continental promontory in central Italy is inserted (models CM, A, D1, D2, D3, D4, and E) and/or the Thyrrenian passive margin of the Adria plate is curved (Model F1);
3. The presence of both structural heterogeneities within the Adria plate (promontory and thin margin) helps the retreat of the Ionian slab till the present‐day position in the Calabro‐Peloritan region.

### 4.3 Comparison With Observed Seismic Anisotropy

Figure 7 shows a comparison between predicted and observed (by Becker et al., 2012) SKS splitting measurements in terms of azimuth and time errors. More in detail, panel “a” shows the observed measurements color‐coded by the error in the orientation compared with synthetic predicted from Model CM at ∼20 My (green bars). The general pattern of the synthetic 3D anisotropy calculations matches the observed data in the whole study region with an average misfit angle of about 26°, but lower values are found in the Calabrian arc (0–10°), southern France (0–20°) and eastern Alps (0–10°), although the mantle flow in the latter area could be affected by its proximity to the model edges. In contrast, a significant mismatch between predicted and observed fast azimuths is found in the Dinaric Alps and Central Apennines where the predictions mainly exhibit a trench‐parallel preferential direction with an average angle rotated by 90° with respect to the observations. These discrepancies could arise from the fact that in this study we have ignored the presence of the Alpine and Dinaric slabs. In fact, the Adria microplate is subducting on both the western and eastern margins, which may cause a strong interaction between the two subduction zones (Király et al., 2018) that has not been considered in our models. The comparison between observed and predicted data is also limited by the fact that some areas are not covered by real measurements as in the case of the Tyrrhenian Sea, the Ionian Sea, and partly in the Central Apennines.

Bearing in mind these discrepancies, our results (Figures 7, 8, 9 and S2) reveal the dominant role of subduction‐induced mantle flow in generating the observed seismic anisotropy and shear wave splitting. Moving from West to East, three main areas characterized by different anisotropy patterns are identified.

Around the area corresponding to the Liguro‐Provençal basin the synthetic SKS splitting, P‐wave anisotropy, and Rayleigh wave azimuthal anisotropy measurements exhibit a smooth ∼90° rotation, with a dominance of NW orientation in the north (southern France) turning progressively toward EW in the Corsica‐Sardinia block. We confirm the interpretation proposed by Lucente et al. (2006) that relates this feature to the formation of the Liguro‐Provençal basin and to the clockwise rotation of the Corsica‐Sardinia block.

In the modeled Tyrrhenian domain, the fast shear wave component orients parallel to the stretching direction of the back‐arc basin, which is consistent with the observations (Jolivet et al., 2009; Lucente et al., 2006). However, delay times are higher (δt=2−3.4s) due to the thick and homogeneous anisotropic layer with sub‐horizontal fast axis extending down to the base of the upper mantle. This might indicate that either dislocation creep is active down to only 200–250 km (Karato & Wu, 1993) or that mantle fabrics are weaker than those modeled.

Beneath the Apennines, the pattern of observed SKS fast axes abruptly changes orientation and becomes parallel to the mountain chain, following its curvature from NW‐SE in the north, to a more NS orientation along the Apennines foreland and to NE‐SW in the farther south Calabria. Along the northern Apennines (Figure 9), the trench‐perpendicular P‐wave anisotropy measured by Hua et al. (2017) could be interpreted with the presence of a large gap in the sinking Ionian slab as in the latest stages of Model CM (Figures 9b and 9d). At earlier stages of Model CM, instead, that is, during or right after slab detachment, the SKS‐splitting delay times are more consistent with the observations, but the P‐wave fast azimuths are trench‐parallel (Figures 9a, 9c and 9e). Because of the striking difference between these two patterns, it follows that a more detailed characterization of the seismic anisotropy in the northern Apennines could provide useful information to determine whether the slab is continuous or not in the upper mantle. In the Sicily channel, smaller‐scale features show trench‐perpendicular SKS azimuths at the western corner of the Calabrian slab, where SKS fast axes turn to NS and then EW in the Tyrrhenian Sea. According to our models, these patterns reflect the retreat of the Adria‐Ionian trenches and related trench‐parallel extension in the sub‐slab mantle, and toroidal flow around the Calabria slab (Civello & Margheriti, 2004; Faccenna et al., 2007, 2014; Jolivet et al., 2009). In our reference Model CM, the SKS fast azimuths are somewhat trench‐perpendicular beneath Central Adria as a result of the mantle flowing through the slab window. However, the scarcity of splitting data in this region does not allow to safely infer the mantle flow at depth and compare our results with the few available observations.

## 5 Conclusions and Outlook

We combined macro‐scale geodynamic modeling with micro‐scale simulations of strain‐induced upper mantle fabrics and seismological synthetics to reproduce and constrain the tectonic evolution of the Central Mediterranean over the last ∼20–30 Myr. Starting from different tectonic scenarios, we have employed 3D thermo‐mechanical simulations to test the role of lithospheric structural heterogeneities and certain rheological parameters on the self‐consistent model evolution. After calculating the upper mantle elastic properties as a function of the strain history and local P‐T conditions, the model results were then validated by comparing seismological synthetics (isotropic P‐wave anomalies, P‐wave anisotropy, SKS splitting, and Rayleigh azimuthal anisotropy) and major tectonic features (i.e., slab and trench geometry) with observations.

Our modeling results bring new insights into the complex structure of the upper mantle beneath the region confirming that the main geological and geophysical observables in the Central Mediterranean can be directly linked to the recent dynamics of the Ionian slab and Adria plate. Furthermore, our results suggest that lateral variations in lithosphere thickness and stiffness can substantially influence the tectonic history of a natural environment. We show that the presence of structural heterogeneities within the Adria plate as imaged by, for example, Miller and Piana Agostinetti (2012) and Panza et al. (2003), and/or the geometry of its Tyrrhenian passive margin plays a fundamental role on:

1. The curvature of the Northern Apenninic trench and its retreat to the Adriatic Sea. This is favored by the presence of the thin continental lithosphere in the northern Adria plate that is prone to subduction.
2. The development of a slab window below the Central Apennines separating two high‐velocity anomalies as shown by seismic tomographies. A good fit between predicted and observed slab morphology was obtained for the models where a thick continental promontory lithosphere is inserted inside the Adria plate. In these scenarios, slab break‐off occurs beneath the area corresponding to the Central Apennines and is favored when the thick promontory is bounded by lithospheric portions (the thin continental margin in the North and the oceanic Ionian plate in the South) that tend to subduct and retreat eastward. This rupture rapidly propagates laterally, allowing, in only a few million years, the formation of two separated arcs, the northern Apennines and the Calabrian arcs. Results inconsistent with tomography observations were instead obtained for the models where the absence of the stiffer Adria promontory results in the complete break‐off of the Ionian slab.
3. The retreat of the Ionian slab till the present‐day position in the Calabro‐Peloritan region in models when both structural heterogeneities were present.

Despite some discrepancies due to the imposed model geometry and some limitations in the methods, the good correlation between predicted and observed slab morphology and seismic anisotropy patterns pose new important constraints on the recent evolution of the study area. Our geodynamic models explain the rotation of the Sardinian‐Corsican block, the opening of the Liguro‐Provençal and Tyrrhenian basins, the retreat of the Apennines and Ionic trenches until their present‐day position. In particular, this work offers valuable constraints on the hypothesized break‐off of the slab beneath the Central Apennines widely debated in the literature, giving a combined geodynamic and seismological reading. Our study demonstrates that this type of combined simulation is able to capture a first order of the overall evolution and the current geological situation of the region providing an overview on the processes that have led to the opening of the Central Mediterranean.

To obtain a better comparison between the synthetic and observed data and to limit the model uncertainties, we envisage that future numerical studies should attempt to (a) improve the model geometry by considering the Earth's sphericity and the Africa‐Eurasia plates convergence, (b) account for the presence of the Alpine and Dinaric slabs that likely affect the mantle flow below the Adria plate surrounding regions, and (c) perform further seismological synthetics, such as P‐wave anisotropic tomographic inversions (e.g., VanderBeek & Faccenda, 2021). At the same time, more abundant and high‐quality seismological measurements could provide more constraints on the upper mantle structure and flow dynamics. For example, at present, it is not yet clear whether the northern Apenninic slab is continuous down to the transition zone or not. Furthermore, SKS splitting in some specific areas such as the Central‐Southern Apennines are sparse, such that there it is not possible to infer whether the shallow upper mantle is flowing through the Central Apennines Slab Gap as a result of the recent retreat of the nearby Calabrian and Northern Apennines slabs. Similarly, P‐wave and S‐wave anisotropic tomography models of the entire Mediterranean region would likely help in better constraining the recent dynamics and deep structure of this region. However, a single P‐wave anisotropic tomography for the Alpine region (Hua et al., 2017) has been produced so far Hua et al. (2017), which is unexpected given the good azimuthal coverage of the area (e.g., Piromallo & Morelli, 2003).

## Supporting information

Supporting Information S1

Movie S1

Movie S2

## Data Availability

T. Gerya provided the I3MG code used for the subduction modeling. This code is available on Figshare (https://figshare.com/articles/software/I3MG/16707370). B. P. VanderBeek created the map in Figure 1b using the software GMT. The modified version of the D‐Rex code used for the fabric modeling, and the routines used to calculate SKS splitting parameters, P‐wave and Rayleigh wave anisotropy can be found inside the ECOMAN software package (https://newtonproject.geoscienze.unipd.it/ecoman/). The MATLAB toolbox geomIO used to define the geometry of the model initial setup can be found at https://geomio.bitbucket.io/. Paraview was used for graphic visualization of the model output (https://www.paraview.org/). Files for visualization in Paraview of the reference Model CM are available on Figshare (https://figshare.com/articles/dataset/File_for_visualization_in_Paraview/16438797). The map in Figure 1b is created with GMT 5.4.3 which is under a GNU Lesser General Public License. Data from real SKS splitting measurements were taken from (Becker, 2006) (https://www‐udc.ig.utexas.edu/external/becker/sksdata). Reviews by Simone Pilia and Luca De Siena have substantially improved an earlier version of this article.
