# Supplementary material for: The Role of Adria Plate Lithospheric Structures on the Recent Dynamics of the Central Mediterranean Region
Source: J Geophys Res Solid Earth. 2021 Oct 8;126(10):e2021JB022377. doi: 10.1029/2021JB022377 (PMC9285053; doi:10.1029/2021JB022377)
Supplement: Supplementary file 1 — Supporting Information S1 [file JGRB-126-0-s001.pdf]

[JGR – Solid Earth]

Supporting Information for

# The role of Adria plate lithospheric structures on the recent dynamics of the Central Mediterranean region

Rosalia Lo Bue[1], Manuele Faccenda[1], Jianfeng Yang[1]

[1] *Dipartimento di Geoscienze, Università di Padova, via Gradenigo 6, Padova, Italy*

**Additional Supporting Information (Files uploaded separately)**

Captions for Supplementary Movies S1 to S2
Supplementary Figure S1
Supplementary Figure S2

**Supplementary Movie S1.** Model CM evolution. Initial setup is shown in Figure 2. In blue the subducted slab (contour at T=1573 K) below ~130 km depth. The continental plates of Adria, Africa and Iberia and the oceanic plate were opacified for better visualization of the subducted slab. The arrows indicate the velocity field at ~250Km depth, and their length is proportional to the velocity magnitude.

**Supplementary Movie S2.** Model F1 evolution. Initial setup is shown in Figure 6a. Color-coding as in Supplementary Movie S1.

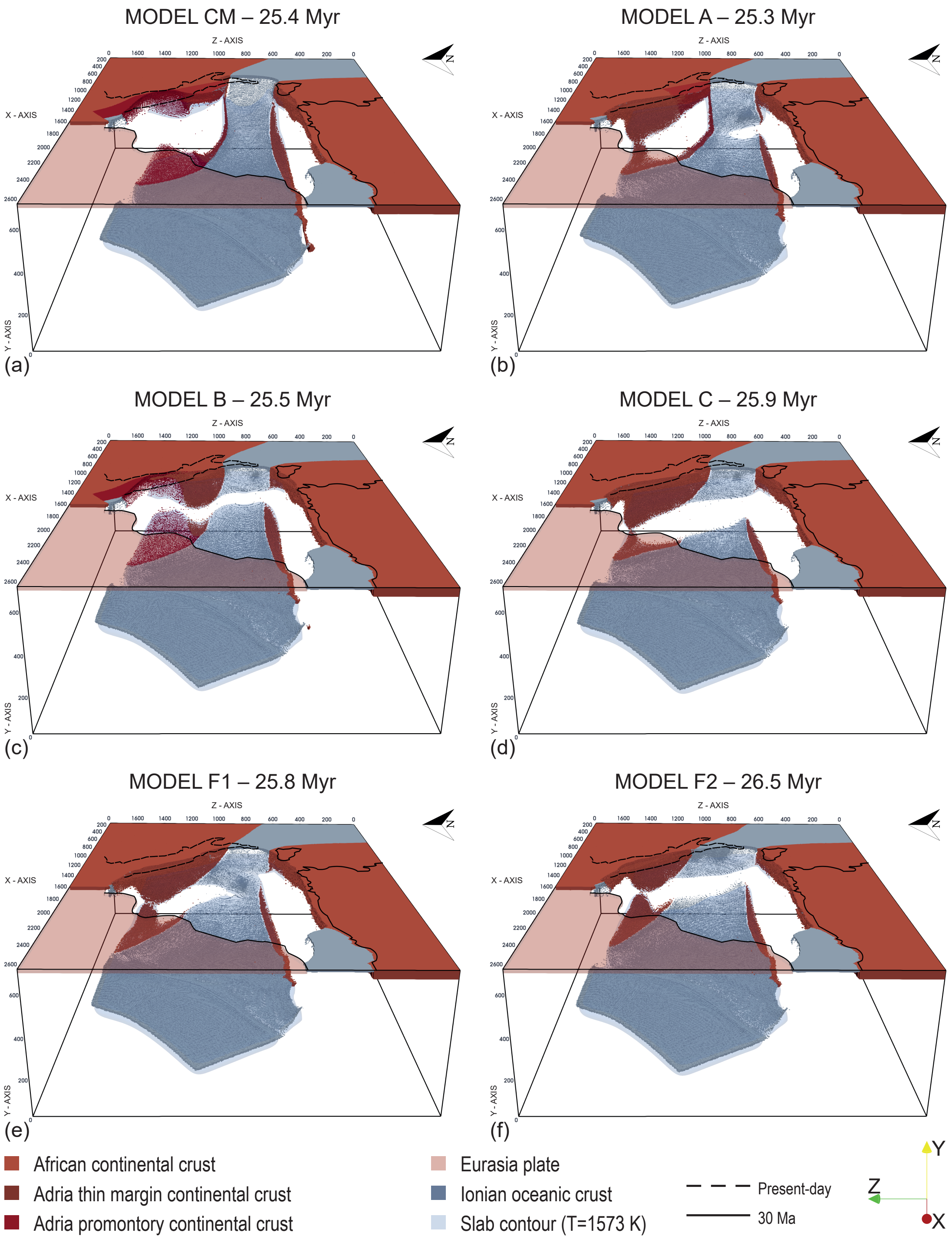


**Supplementary Figure S1.** Snapshot at ~25Myr of: (a) Model CM, (b) 5Model A, (c) Model B and (d) Model C, (e) Model F1 and (f) Model F2. Color-coding and coastlines as in Fig. 3.

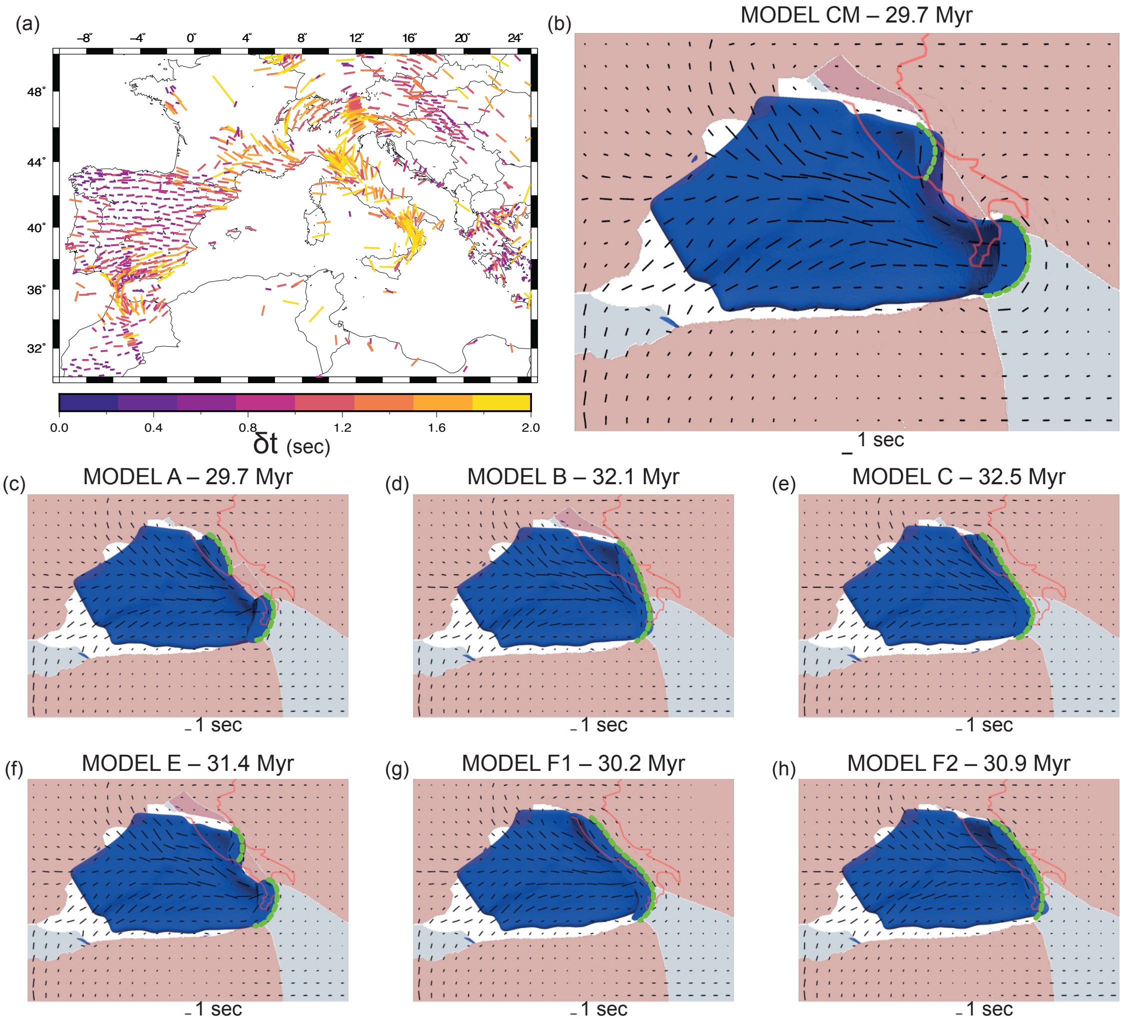


**Supplementary Figure S2.** (a) SKS-splitting measurements in the Central-Western Mediterranean (as in Fig. 1b) compared with synthetic SKS splitting measurements for: (b) Model CM, (c) Model A, (d) Model B, (e) Model C, (f) Model E, (g) Model F1 and (h) Model F2. The EW black bars outside the domain indicates 1 sec. The blue surface envelopes the subducted lithosphere (T < 1573 K) below 120 km depth. The present-day coastlines of peninsular Italy are indicated in red for reference. The green dashed thick line indicates the position of the active trenches.
